# Allosteric Enzyme-Based Biosensors—Kinetic Behaviours of Immobilised L-Lysine-α-Oxidase from *Trichoderma viride*: pH Influence and Allosteric Properties

**DOI:** 10.3390/bios10100145

**Published:** 2020-10-17

**Authors:** Antonio Guerrieri, Rosanna Ciriello, Giuliana Bianco, Francesca De Gennaro, Silvio Frascaro

**Affiliations:** 1 Dipartimento di Scienze, Università degli Studi della Basilicata, Viale dell’Ateneo Lucano 10, 85100 Potenza, Italy; rosanna.ciriello@unibas.it (R.C.); giuliana.bianco@unibas.it (G.B.); 2 Bio Basic Europe, Via Panizzi Antonio 10, 20146 Milano, Italy; francesca-dg@live.it; 3 RBM S.P.A.—Istituto di Ricerche Biomediche “A. Marxer”, Via Ribes 1, 10010 Colleretto Giacosa (To), Italy; silvio.frascaro@gmail.com

**Keywords:** amperometric biosensor, biorecognition kinetics, L-lysine-α-oxidase, Trichoderma viride, immobilised enzyme, allosteric behaviour, pH effect

## Abstract

The present study describes the kinetics of L-lysine-α-oxidase (LO) from *Trichoderma viride* immobilised by co-crosslinking onto the surface of a Pt electrode. The resulting amperometric biosensor was able to analyse L-lysine, thus permitting a simple but thorough study of the kinetics of the immobilised enzyme. The kinetic study evidenced that LO behaves in an allosteric fashion and that cooperativity is strongly pH-dependent. Not less important, experimental evidence shows that cooperativity is also dependent on substrate concentration at high pH and behaves as predicted by the Monod-Wyman-Changeux model for allosteric enzymes. According to this model, the existence of two different conformational states of the enzyme was postulated, which differ in Lys species landing on LO to form the enzyme–substrate complex. Considerations about the influence of the peculiar LO kinetics on biosensor operations and extracorporeal reactor devices will be discussed as well. Not less important, the present study also shows the effectiveness of using immobilised enzymes and amperometric biosensors not only for substrate analysis, but also as a convenient tool for enzyme kinetic studies.

## 1. Introduction

Enzymes, particularly oxidoreductases, are common biorecognising components of electrochemical biosensors, mainly due to their high selectivity and ability to convert scarcely detectable analytes into electroactive substances. Clearly, the biosensor response is mainly controlled by enzyme kinetics, even if complicated by substrate mass transfer through the immobilised enzyme layer. Despite this, the effect of enzyme kinetics on biosensor operation is often overrated or, worse, neglected. Indeed, valuating and improving their analytical performances, the classical Michaelis-Menten model is quite often used to fit their responses, which is not necessary the best approach for response modelling and optimisation; moreover, the selectivity issue is usually approached by selecting the enzyme from specific sources and/or using particular immobilisation procedures, while a thorough study of its kinetics could provide useful insights for this focus. Last but not least, biosensors are not only devices for substrate analysis, but also a convenient tool for enzyme kinetic studies. With respect to the classical approach in homogeneous phase, in fact, amperometric biosensors give all the well-known advantages of using immobilised enzymes (e.g., low enzyme consumption for their studies since reusable many times) while allowing to access a great amount of kinetic data in a short amount of time. More importantly, the product of the enzymatic reaction does not accumulate (since it is quickly consumed or removed from the immobilised system under study), preserving the initial velocity measurements required for a correct kinetic analysis.

L-lysine-α-oxidase (LO) is an oxidoreductase that catalyses the oxidation of the well-known essential amino acid L-lysine (Lys) according to the following reaction:

L-lysine + O_2_ → α-keto-ε-aminocaproate + H_2_O_2_ + NH_3_

where the produced α-keto-ε-aminocaproate successively dehydrates spontaneously to Δ^1^-piperidine-2-carboxylate. The enzyme was firstly isolated from *Trichoderma viride* [1,2], but soon was found also in other Trichoderma species and strains [3,4,5,6,7,8] and characterised as well (see refs. [9,10] for a review). LO is an L-amino acid oxidase that is almost specific to Lys, but, depending on the enzyme source, other Lys analogues (such as L-ornithine, L-tyrosine and L-arginine) are oxidised too to some extent—no matter the analogues, it is the α-amino group of the amino acid the residue that is oxidised by the enzyme, while the terminal amino group appears to be important in the binding of amino acids to the enzyme and/or to the enzyme catalysis [2]. Furthermore, LO shows a molecular weight of about 110–116 kD (depending on the source) [2,4,5,6,7,8,9,10] and consists of two subunits of the same molecular weight of about 55–58 kD containing one FAD per subunit [2,4,5,6,7,8,9,10].

The main interest in LO comes from its peculiar biological properties and its likely applications in medicine. In fact, LO was discovered through the evidence that the aqueous extract of a wheat bran culture of *Trichoderma viride* from soil contains a new antitumor substance of high molecular weight [1,2]. From that evidence, further biological effects, both in vitro and in vivo, were discovered (see refs. [9,10] for a thorough discussion). For example, LO appears to suppress DNA, RNA and protein synthesis in leukaemia, human ovary carcinoma and lymphoma tumour cells in vitro but antileukemia and antimetastatic effects in rather small effective doses have been confirmed in vivo too. Unfortunately, the practical application of LO in oncology might induce undesirable side effects since, e.g., the injection of L-asparaginase preparations (the unique enzyme employed in cancer therapy until now), is well known to cause anaphylactic shocks; in this respect, using immobilised LO as an extracorporeal shunt, so as to avoid the access of the enzyme into the organism, has been suggested [11]. Obviously, the application and use of a similar extracorporeal medical device require a successfully LO immobilisation and a thorough study of its catalytical behaviour as immobilised enzyme.

Until now, the main application of immobilised LO is instead for Lys assay. Indeed, several Lys biosensors have been described, and in the authors’ laboratory, a novel, highly selective LO (from *Trichoderma viride*)-based amperometric biosensor has been developed, which proved useful and effective for the selective Lys analysis in pharmaceutical and food samples, and in untreated human serum as well [12,13,14,15] (see those references for updated information about the Lys biosensors described thus far and for a critical comparison). Particularly, the selectivity problem of the Lys assay required a careful study and control of the kinetic behaviour of the sensing device and, interestingly, during those studies, unexpected anomalies in LO enzyme kinetics were observed. For example, on increasing the working pH, an ever-increasing deviation from the classical Michaelis-Menten model of enzyme catalysis was reported [12], and unusual hydrodynamic behaviours were observed as well [12,13]; more recently, a kinetic study of immobilised LO from *Trichoderma viride* evidenced the allosteric behaviour of this enzyme [15], thus confirming the allosteric effects observed for LO from other sources (from Trichoderma cf. aureoviride Rifai VKM F-4268D), even if this latter kinetic studies were performed in solution [16]. Naturally, all these deviations from the classical model of enzyme catalysis require a thorough deepening if this enzyme need to be used in Lys assays and medical therapy.

In the present study, we will look further into the kinetic of immobilised LO from *Trichoderma viride* showing that its allosteric behaviour is strongly dependent on pH—the implications regarding the ionic form of Lys involved in the allosteric behaviour of the enzyme will be presented and discussed as well. Furthermore, we will show that the Monod-Wyman-Changeux (MWC) rather than the well-known Hill allosteric model, as previously used elsewhere [15,16], appears to be the best approach in describing the cooperative binding of LO. Finally, some considerations about the influence of the peculiar LO kinetics on biosensor operations and extracorporeal reactor device will be discussed.

## 2. Materials and Methods

### 2.1. Materials

L-lysine, glutaraldehyde (grade II, 25% aqueous solution), bovine serum albumin (fraction V) and L-lysine-α-oxidase (EC 1.4.3.14, from *Trichoderma viride*, 49.31 units per mg protein) were bought from Sigma Chemical Co. (St. Louis, MO, USA). Other chemicals were analytical reagent grade. L-lysine stock solutions were stored at 4 °C. A Britton-Robinson buffer (0.1 M) was used in all the kinetics studies. Double distilled-deionised water was used to prepare all solutions.

### 2.2. Apparatus

The flow injection set-up consisted of a Gilson Minipuls 3 peristaltic pump (Gilson Medical Electronics, Villiers-Le-Bel, France) and a seven-port injection valve (Rheodyne mod. 7725, Cotati, CA, USA) equipped with a 20 µL sample loop. The electrochemical detector was an EG&G Model 400 (Princeton Applied Research, Princenton, NJ, USA) including a thin-layer electrochemical cell with a Pt disk (3 mm diameter) working electrode and an Ag/AgCl, 3 M NaCl reference electrode; two thin layer flow cell dual gaskets (Bioanalytical Systems, Inc., West Lafayette, IN, USA) of 0.004-inch thickness were used. The sample injection valve was connected to the electrochemical detector by a PEEK tubing (0.25 mm ID, 130 cm length). Flow injection signal chart recording was achieved by a Kipp & Zonen (Delft BV, Holland) mod. BD 11 E Flatbed Yt recorder.

### 2.3. Methods

#### 2.3.1. Enzyme Immobilisation and Biosensor Preparation

The enzyme was immobilised onto a Pt disk working electrode. The Pt surface was preliminary cleaned by few drops of hot nitric acid, washed with bidistilled water, then polished to a mirror finish by alumina (0.05 µm particles) and finally extensively washed and sonicated in bidistilled water.

L-lysine-α-oxidase (LO) was immobilised and L-lysine (Lys) biosensors were prepared as follows [15]. Briefly, 26.3 units of LO were dissolved into 250 µL of phosphate buffer (pH 7.4, 0.1 M). Then, 2.6 mg of bovine serum albumin was dissolved in 50 µL of the enzyme solution and carefully mixed with 5 µL of 5% glutaraldehyde solution (25% glutaraldehyde solution diluted 1:5 with phosphate buffer pH 7.4, 0.1 M). Four µL of the resultant solution were carefully pipetted onto the Pt disk working electrode surface and meticulously spread out to cover completely the electrode surface avoiding air bubble formation; after that, the modified electrode was left to crosslink and air-dry at room temperature for a few minutes. The resulting Pt/LO modified electrode was preliminarily soaked in the background electrolyte for a few minutes before its first use to allow the removal of adsorbed or weakly bound enzyme from the enzyme membrane as well as for its swelling. The biosensor was stored in phosphate buffer, pH 7.4, 0.1 M, at 4 °C in the dark when not in use.

#### 2.3.2. Electrochemical Measurement

A detection potential of +0.7 V versus Ag/AgCl/NaCl (3M) was used in all the flow injection electrochemical measurement, as the lowest potential value showing maximum sensitivity and pH independence towards hydrogen peroxide oxidation and, hence, towards Lys detection. The Lys injection volume was 20 µL, solutions and carrier stream were air saturated and the temperature was ambient (20 °C).

## 3. Results

### 3.1. Framework of Kinetic Measurements

L-lysine-α-oxidase from *Trichoderma viride* (LO) was co-immobilised with an inert protein, bovine serum albumin, by crosslinking with glutaraldehyde. This valuable co-crosslinking immobilisation approach [17] allowed us to obtain an immobilised enzyme layer with high biocomponent activity and good stability properties by simply casting a small amount of the proper co-crosslinking enzyme solution onto the electrode surface [18]. Of course, the influence of the inert protein and crosslinker concentrations have strong impact on the efficiency of LO immobilisation and its catalytic properties and have been already studied and optimised elsewhere [12].

**Figure 1:**
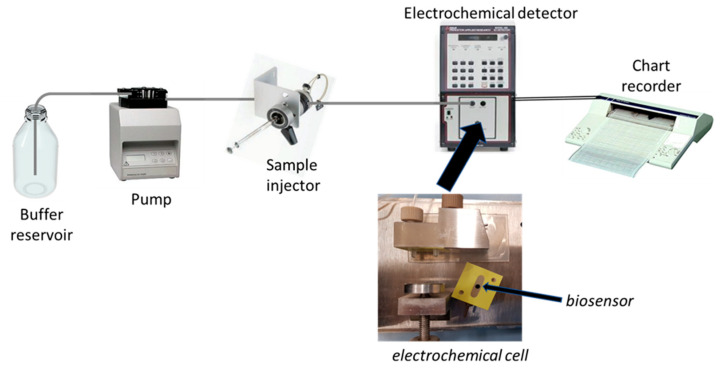
Schematic diagram of the flow injection setup used in the kinetic measurements showing the electrochemical cell and the relevant enzyme modified electrode (biosensor) used in the amperometric measurements.

**Figure 2:**
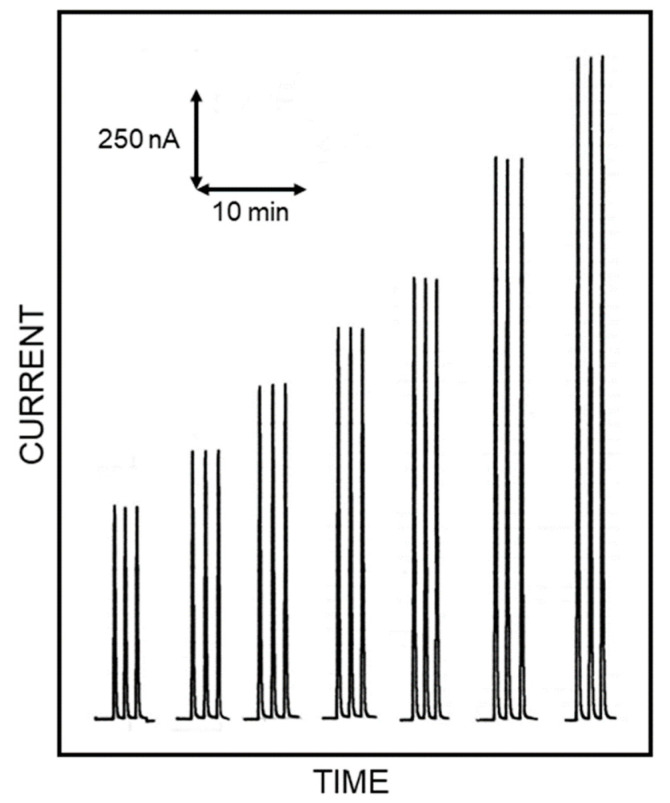
Typical flow injection peaks at a Pt/LO biosensor due to triplicate injections of L-lysine standard solutions at levels of (left to right) 1, 1.25, 1.5, 1.75, 2, 2.5 and 3 mM. Carrier stream: pH 5, flow rate 1 mL min^−1^. Other experimental conditions are described in the Materials and Methods section.

Accordingly, this procedure permitted us to realise an amperometric enzyme electrode showing high sensitivity and fast response time, with an enzyme layer so stable under stirring or flowing solutions to permit a flow injection analysis of L-lysine (Lys) sample, and thus, a kinetic study of the immobilised enzyme (see Figure 1 for a schematic diagram of the flow injection setup used in the kinetic measurements). In fact, the flow injection system permitted here to study the kinetics of the immobilised enzyme in a simple straightforward way as demonstrated by Figure 2, showing flow injection responses due to repeated injections of standard Lys solutions, acquired in optimal conditions for this study. Indeed, the responses were practically identical for each Lys level demonstrating the high repeatability of the present approach (within-a-day coefficients of variation for replicate (*n* = 5) Lys injections were 0.92% and 1.35% at 4 mM and 0.2 mM Lys levels, respectively); the sensor-to-sensor repeatability was also tested by fabricating three different biosensors on different days. In the worst case scenario, a response deviation of 4.6% (at 1.25 mM Lys level) was observed, demonstrating the good repeatability of biosensor production. Moreover, the amperometric enzyme electrode here used showed a linear range of almost three decades [13], permitting a limit of detection (at a signal to noise ratio of 3) as low as 1 µM in batch experiments [12], whereas in flow injection analysis it was 4 µM [13] (corresponding to a limit of quantification of 13 µM). Furthermore, fast responses were also observed due to the low response time of the biosensor here used [12], which allowed a high sample throughput (less than 1 sample min^−1^) and permitted us a complete kinetic study in a few minutes, thus minimising any undesired side effects due to, e.g., long-term variations of enzyme activity or substrate permeability in membrane. Not less important, the flow injection setup showed a rapid start-up time (typically less than minutes), no drift in signal measurements while allowing very low sample sizes (typically 20 µL) and low carrier consumption.

In this respect, Scheme S1 in the Supplementary Materials represents a figurative illustration of the amperometric enzyme electrode used in the kinetic measurements, pointing out the enzyme catalysis and the relevant mass flows involved (see ref. [19] for a further deepening about). Here, Lys (*S*) reacts with the enzyme LO (*E*) immobilised in the enzyme membrane producing hydrogen peroxide (*P*), which is easily detected (by electrooxidation) at the electrode surface and generates a current that is proportional to the amount of hydrogen peroxide produced by the enzyme. Naturally, this permitted us to follow the kinetic of LO in a simple way while the coupled flow injection setup allowed for easy and fast substrate sampling. With respect to the classic kinetic in solution, however, here, the enzyme kinetic is coupled (from left to right in Scheme S1) to the electron transfer kinetics, diffusion of the substrate/product in membrane and to the mass transfer of the substrate supplying solution (for sake of simplicity, any partitioning or electrostatic effects coming from enzyme membrane have been here considered); of course, these mass transfers involve a substrate concentration gradient both in membrane and in solution which is roughly shown by shading in Scheme S1. Finally, the effect of dioxygen on the LO enzymatic reaction was here neglected, since it has been demonstrated that its influence is reduced when dioxygen is supplied by the electrolyte solution and its diffusion is limited through the enzyme membrane [20] as in the present case. Nevertheless, in the present study, air-saturated solutions were used both for carrier stream and standards to further limit any dioxygen dependence. Indeed, the highly within- and between-days repeatability (see above) indirectly confirmed the dioxygen independence.

The electrooxidation of hydrogen peroxide at the electrode surface is very fast, and thus never limiting; furthermore, it is always proportional to the hydrogen peroxide concentration, whatever its concentration or pH, thus, nonlinear behaviours due to electron transfer kinetics are unlikely. Accordingly, no build-up of ***P*** in membrane is expected, and quickly after the substrate supply, a steady-state condition can be assumed, being limited by enzyme kinetics and/or substrate mass transfer. The “external” substrate mass transfer (i.e., that coming out from the substrate supplying solution) can be easily increased by increasing the flow rate in the flow injection measurements but reaches a limiting value due to the membrane thickness, since no convective flow is possible within the membrane [21]; hence, in these studies, flow rate is an optimal tool to switch between an external diffusive control through solution (low flow rates) to a diffusive, limiting control through the membrane (high flow rates). Conversely, the “internal” substrate mass transfer (i.e., substrate diffusion in the enzyme entrapping membrane) is fixed depending on the substrate permeability of the used membrane, the latter of which can be limiting for fast enzyme kinetics or, vice versa, limited by enzyme kinetics for poor enzyme activity; since pH usually modulates the activity of the enzymes, changing the pH on the enzyme kinetic studies is another helpful tool to switch between enzyme or limiting diffusion kinetics. Please note that even under substrate mass transfer limiting conditions, the kinetic measurements reflect the catalytic enzyme behaviour since these limiting conditions simply involve an apparent dilution of substrate concentration.

### 3.2. pH Dependence of the Allosteric Behaviour of the Enzyme

The influence of pH on the activity of the LO enzyme as immobilised in the present study has been already reported elsewhere [12,13]. Briefly, the activity of the immobilised enzyme (studied in a pH range of 5–9.5 and measured as the sensitivity to the Lys response) increased the pH, reaching a maximum and nearly levelling off from pH 7.5. This behaviour agrees with that already reported for the native enzyme in solution [2], indicating that the entrapped membrane and the ammonia enzymatic production and hydrogen peroxide electrooxidation in the membrane do not significantly influence the ionisation processes of both substrate and enzyme in membrane; furthermore, pre-incubation studies ruled out any potential changes in enzyme stability in that pH range [12]. Remarkably, the dependence of the apparent Michaelis-Menten constant *K*’_M_ with pH behaved similarly but in a specular fashion, i.e., *K*’_M_ decreased with pH till about pH 7 (from about 3.5 mM down to nearly 1.7 mM, while a 0.04 mM value was reported for the native enzyme [2]), which then remained constant up to pH 9.5 [12]. Lys shows its isoelectric point (p*I*) at pH 9.7, while its p*K*_a_ values are 2.2, 8.9 and 10.3; accordingly, the formation of a proper Lys ionic form to explain the observed enzyme activity increase in the pH range 5–7.5 should be ruled out, since the diprotic Lys form is already the main Lys specie in that pH range (see Scheme S2 in Supplementary Materials). On the other hand, both the pH behaviours previously described are in agreement with competitive inhibition kinetics [22] by H^+^ ions. After increasing the pH, the enzyme molecules are converted from the inactive, enzyme-inhibitor (E-H^+^) complex, dead-end form to the full affinity form (E); thus, enzyme activity increases and *K*’_M_ decreases. LO has its p*I* at pH 4.35 [2] so that the active site of LO loses a proton to bind Lys and catalyse its oxidation; due to the pH range in which the enzyme activity increase is observed, the deprotonation of the side chain of an histidine residue may be hypothesised here (p*K*_a_ values 1.77, 6.10 and 9.18), considering that about 24 histidine residues have been found in LO [2] and that histidine plays an active role in the mechanism of many enzyme reactions [23]. Indeed, a histidine residue has been invoked in L-amino acid oxidase mechanism, acting as a base to catalyse proton removal from L-leucine [24,25].

**Figure 3:**
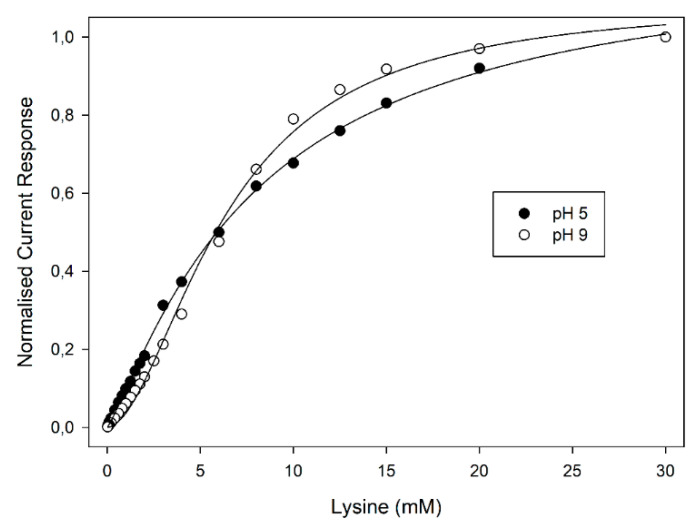
Normalised current responses due to injections of L-lysine standard solutions at the amperometric enzyme electrode as a function of L-lysine concentration at pH 5 (•) and pH 9 (o). Continuous lines refer to Hill fitting of data. Flow rate: 1 mL min^−1^; other experimental conditions are described in the Materials and Methods section.

As pointed out elsewhere [15,16], kinetics studies of LO from *Trichoderma viride* and from *Trichoderma* cf. *aureoviride* Rifai VKM F-4268D showed a notable allosteric behaviour of these enzymes; in any case, those studies were performed at fixed pH values and the influence of pH on the cooperative behaviour of the enzyme was never explored. Accordingly, the pH influence on the enzyme kinetics was here studied using a Britton-Robinson universal buffer (i.e., acetate/phosphate/borate) at fixed ionic strength (0.1 M), to avoid any change in the ionic composition of the supporting electrolyte. As can be seen from Figure 3, reporting the normalised current responses of the amperometric enzyme electrode vs. Lys concentration at two pH values for the sake of clarity, the plots display the well-known kinetic behaviour expected for enzyme catalysis (i.e., linear and saturated responses at low and high substrate concentrations, respectively). A critical inspection of Figure 3 showed a significant deviation from the expected hyperbolic behaviour (i.e., Michaelis-Menten model) to a sigmoidal-like plot as the pH increased; furthermore, these deviations were particularly evident starting from about pH 7 and reached their maximal deviation at about pH 9. The sigmoidal behaviour of LO kinetics of course confirmed the allosteric behaviour of this enzyme. Indeed, as pointed out in the Introduction section, LO is a dimeric enzyme consisting of two identical subunits each one containing a FAD unit [2,4,5,6,7,8,9,10], thus, cooperative binding could arise; nevertheless, a pH dependence of the cooperation was never reported before and accordingly a deeper study was performed. Please note that the pH dependence on the allosteric behaviour of enzymes is not surprising, since it was already described in 1904 by Christian Bohr while studying the oxygen binding affinity of haemoglobin, a striking pH effect well-known as the Bohr effect (see for example ref. [26] for some historical nods and the relevant modelling).

The modelling of an allosteric enzyme is rather intricate. Frequently, as a first approach, the Hill equation is used for the kinetics of such enzymes [22]. In this case, it can be proven that for an allosteric enzyme with *n* equivalent subunits (2 for the present enzyme), the velocity equation *v* is:

$$v=\frac{V_{\max}\cdot {\left[s\right]}^{n}}{\left(K_{0.5}^{n}+{\left[s\right]}^{n}\right)}$$

where [*s*] is the substrate concentration, *V*_max_ the maximum velocity and *K*_0.5_ the substrate concentration at *V*_max_/2 (which reduces to *K*_M_, the Michaelis-Menten constant, when *n* = 1). Please note that the Hill approach is strictly valid for high cooperativity [22]: yet, where cooperativity is not so high, enzyme kinetics can be even described by Hill-type equations but the Hill coefficient, *n*, corresponding to the number of substrate binding sites per enzyme molecule (2 in the present case) misses its physiochemical sense becoming an apparent Hill coefficient, *n*_app_, still describing the cooperative degree between the active sites of the enzyme, but assuming non-integer values, usually less or at least approaching the actual (integer) number of sites present in the enzyme [22]. Using the Hill model as the first approach in fitting the current responses observed at the amperometric enzyme electrode as a function of Lys concentration gave the *n*_app_ values reported in Table 1 for different pH values and several flow rates. As can be seen, *n*_app_, and hence, the cooperative behaviour of LO, increased with pH increase, starting from about pH 7, and reaching its maximal (expected) value at about pH 9. Naturally, as already pointed out above, the enzyme kinetic is complicated by substrate mass transfer; thus, discrimination between enzyme and mass transfer limitations is essential.

The influence of mass transfer on to the response of the present amperometric enzyme electrode has been already reported elsewhere [12,13]. As those studies showed, increasing the flow rate permitted to maximise the “external” substrate mass transfer until reaching (starting from flow rates higher than 1 mL min^−1^) a limiting value due to the (finite) thickness of the membrane entrapping the enzyme; furthermore, the same studies showed that changing the pH (e.g., changing the activity of the immobilised enzyme) permitted to change from enzyme to “internal” substrate mass transfer limitations at every flow rates. Accordingly, further kinetic studies were performed at different flow rates, and the relevant current responses observed at the amperometric enzyme electrode as a function of Lys concentration were fitted using the Hill model. As Table 1 shows, the *n*_app_ observed at several flow rates but at a fixed pH values were all nearly the same, demonstrating that the cooperative behaviour observed for LO and its pH dependence was not due to mass transfer complications but simply due to enzyme kinetics. The observed non-dependence of *n*_app_ from the flow rate is not unexpected; indeed, mass transfer limitations usually involves variations in *K*’_M_ and the apparent maximum velocity *V’*_max_ in immobilised enzyme systems, due to the dilutions effects raising from those limitations in membrane [19]; on the contrary, *n*_app_ would denote the “equation form” used to describe the enzyme kinetics, i.e., the kinetic mechanisms involved in enzyme catalysis, which are of course independent on substrate supply.

### 3.3. Application of Monod-Wyman-Changeux model

As is well known, the Hill velocity equation can be linearised by converting it on its logarithmic form [22], the well-known Hill plot:

$$\log\frac{v}{V_{\max}-v}=n\log[s]-\log K_{0.5}^{n}$$

As pointed out in many studies, the Hill plot is not a straight line for many allosteric enzymes [26]; apart from demonstrating the simplicity (and weakness) of the Hill model, those deviations from the expected linearity show that, usually, cooperativity binding in an enzyme is not fixed but depends on the saturation of the enzyme, i.e., from substrate concentration, that is, the *n* parameter is substrate concentration dependent.

**Figure 4:**
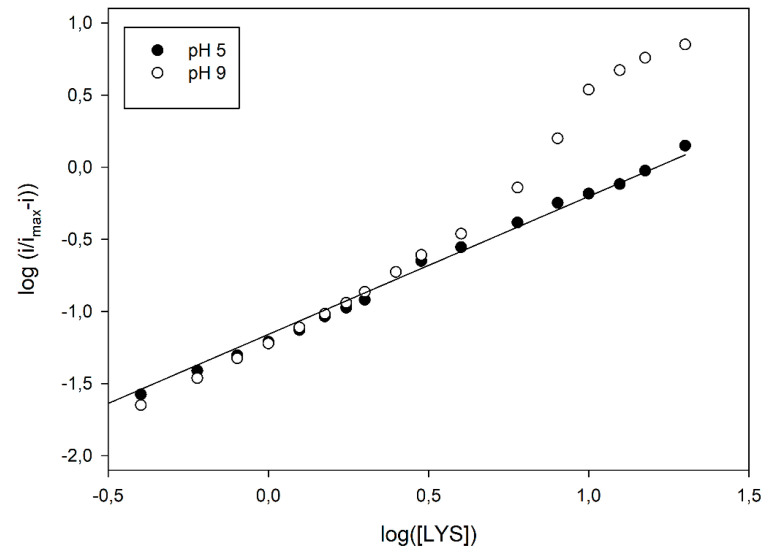
Hill plot of normalised current responses vs. L-lysine concentration as shown in Figure 3. Straight line refers to linear fitting of data points at pH 5 (•).

The first kinetics studies evidencing the allosteric behaviour of LO [15,16] used the Hill equation to demonstrate the departure from the classical Michaelis-Menten approach and calculate the relevant Michaelis-Menten constant. Even in the present study, the Hill equation was preliminary used to show the dependence of the cooperativity on pH; a closer inspection of data compared to those reported in Figure 3 already evidenced a departure from the simple sigmoidal fitting. Indeed, conversion of kinetic data in the relevant logarithmic Hill plots displayed a significant nonlinear behaviour, at higher pH values, as shown in Figure 4; similar deviations were also observed at different flow rates (see Figure S1 in Supplementary Materials), demonstrating once again that the enzyme kinetics and the relevant departures from the Michaelis-Menten model were not artefacts originating from mass transfer limitations. In particular, as shown in Figure 4, a linear Hill plot was observed at pH 5, of which the slope (0.96 ± 0.02, correlation coefficient better than 0.998, three replicates, 17 data points fitted) agreed with the Michaelis-Menten model, as already pointed out in Table 1; on the contrary, striking deviations from linearity were evident from about pH 7, which reached their maximal deviations at about pH 9, where a skewed sigmoidal plot was observed.

From the initial, phenomenological application of Hill equation, several different approaches and models were developed to describe the cooperativity in enzyme kinetics, in an attempt to offer a biochemical vision of the underlying mechanism (see ref. [26] for a review of the relevant approaches developed). In particular, one of the most recent and best approaches describing cooperativity was proposed by Monod et al. [27,28], known as the Monod-Wyman-Changeux (MWC) model. Briefly, in this approach, the allosteric enzyme is modelled as two (or more) interconvertible conformational states, namely the tense (*T*) and relaxed (*R*) states, coexisting in a thermal equilibrium and differing in their affinity for the substrate. The binding of ligand molecules stabilises the higher affinity state, controls the ratio between the two states and induces a change of all enzyme subunits states at the same time, a phenomenon known as “concerted transition”. If the ligand dissociation constants for the *T* and *R* states are $K_{d}^{T}$ and $K_{d}^{R}$, respectively, their ratio *c* = $K_{d}^{R}$/$K_{d}^{T}$ offers a suggestion of the difference of substrate affinities for the two states. Of course, if *c* = 1, the affinities are the same and the MWC model simplifies to the Michaelis-Menten model; on the contrary, if *c* is less than unity, the substrate shows much more affinity towards the relaxed *R* state and, therefore, the equilibrium between the two states shifts towards the *R* state after one substrate binding.

**Figure 5:**
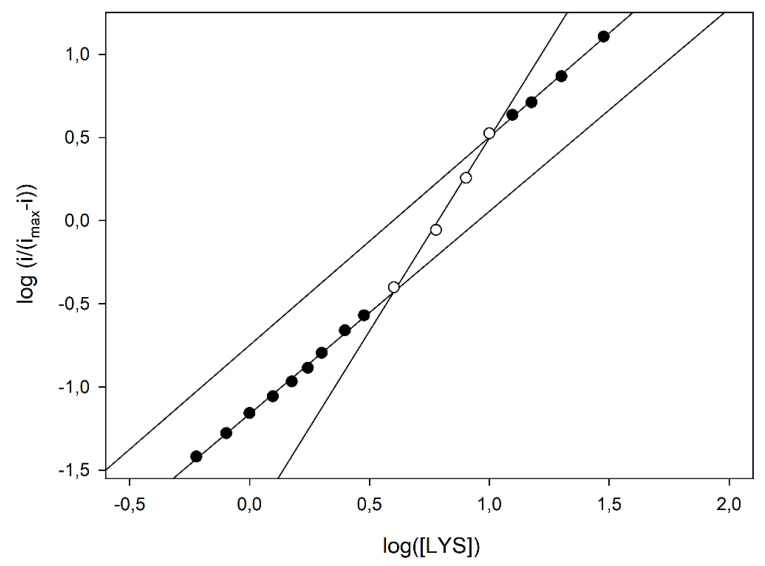
Hill plot of normalised current responses due to injections of L-lysine standard solutions at the amperometric enzyme electrode as a function of L-lysine concentration at pH 9. Straight lines refer to linear fitting of data points (see manuscript for further explanation about). Flow rate: 1.0 mL min^−1^; other experimental conditions are described in the Materials and Methods section.

According to the MWC model, the skewed sigmoidal plots here observed at the higher pH values (as in Figure 4) can be viewed as a progressive transition from the tense, low affinity *T* state to the relaxed, high affinity *R* state as the saturation of the enzyme, i.e., the substrate concentration, increases. In particular, by analysing the Hill plot at pH 9 (see Figure 5), some kinetic data can be inferred using the MWC approach. In fact, the apparent Hill coefficient, *n*_app_, can be estimated by the slope of the curve and, as Figure shows, it starts from an initial value of 1.20 ± 0.03 (left, low substrate concentrations, linear branch, three replicates, nine data points fitted), reaches its maximal value of 2.32 ± 0.13 at the inflexion point, i.e., at about half enzyme saturation (central linear branch, three replicates, four data points fitted) and decreases again to a low value of 1.25 ± 0.05 (right, high substrate concentrations, linear branch, three replicates, four data points fitted). Of course, this is a clear indication that, in the LO enzyme, cooperativity is Lys concentration dependent, since the maximum is at about half enzyme saturation and the minimum is at low and high saturations; accordingly, the *n*_app_ values listed in Table 1, obtained by the simple Hill fitting, could represent a weighed mean value of cooperativity, since the Hill approach does not consider any substrate concentration dependence on cooperativity. Furthermore, the intercept of two asymptotes of the skewed sigmoidal plot (left and right linear branches) and the *y* axis in Figure 5 can permit to estimate the logs of $K_{d}^{T}$ and $K_{d}^{R}$ constants, respectively, and thus, the parameter *c*. By means of a linear fitting (correlation coefficients better than 0.996), logs of $K_{d}^{T}$ and $K_{d}^{R}$ were −1.162 ± 0.006 (three replicates, nine data points fitted, equivalent to about 69 µM) and −1.82 ± 0.11 (three replicates, four data points fitted, equivalent to about 15 µM), respectively, and the ratio *c* about 0.22, demonstrating that by increasing the LO saturation (i.e., the Lys concentration), the equilibrium between the two states shifted towards the relaxed, high affinity *R* state.

According to this point of view, catalytic schemes of LO reactions, such as those pointed out for LO from *Trichoderma* cf. *aureoviride* Rifai VKM F-4268D [16], can be hardly invoked in the present case, since they do not rely with any pH dependence of cooperativity, as is demonstrated in the present study. Since the MWC approach appears to be the best model describing the observed kinetics of LO from *Trichoderma viride*, some considerations and hypotheses need here to be postulated to tentatively explain the presence of two interconvertible conformational states of LO (the tense *T* and relaxed *R* states), their difference in affinity for Lys and their dependence on pH. In this respect, many studies on the oxidation of amines by flavoproteins pointed out that the enzyme mechanism currently accepted involves the removal of a proton from the α-carbon of substrate by a base (the here supposed histidine residue in the present case) and the concerted hydride transfer from the neutral α-amino group of substrate to the FAD group of the oxidised enzyme [25,29]; naturally, if the α-amino group of substrate is in its protonated, acid form $-NH_{3}^{+}$, the relevant hydrogen transfer requires deprotonation, probably mediated by water molecules filling activity site of the enzyme, as they were found in the structure of a bacterial L-amino acid oxidase from *Rhodococcus opacus* [30]. Under this light, the enzyme–substrate complexes with the protonated or neutral α-amino group substrate might differ in their conformational states and affinity for the substrate, originating the cooperativity of LO. To corroborate this point of view and, more importantly, the pH dependence of cooperativity, the distribution diagram of Lys species with pH might help (see Scheme S2 in Supplementary Materials). As can be seen, in the pH range 7–9 (i.e., in the pH range where cooperativity was observed) the diprotic Lys form decreases with pH while the monoprotic form increases; if there is an affinity towards the latter, the neutral α-amino group form is higher with respect to the charged, diprotic form, the pH dependence of cooperativity can be explained in a first instance by the increase of this highly affinity form with pH.

### 3.4. Influence of LO Allostery and Its pH Dependence on Biosensor Performance

Allosteric enzymes appear quite interesting in principle since modulation by effectors or inhibitors on enzyme catalysis are clearly anticipated and can be strategically used for biosensor production [31]. Even in cases where substrate and effector/inhibitor are the same molecule (as in the present case), allostery can be advantageous. Single-site binding enzymes (i.e., Michaelis-Menten kinetics) are characterised by a hyperbolic relationship where the dynamic range is fixed (*n*_app_ = 1); on the contrary, allosteric enzymes or a network of enzymes with different affinities toward the same substrate, due to their different, sigmoidal-like kinetics, can be useful used to extend (*n*_app_ < 1) or narrow (*n*_app_ > 1) the dynamic range of related biosensors [32]. This should extend the usefulness of electrochemical biosensors in applications where the concentration of the analyte can vary over many orders of magnitude or, vice versa, where monitoring of the analyte within a narrow concentration window is required and high sensitivity and a steep relationship are preferred. As Figure 3 shows, in the present case, LO allostery and its pH dependence can be advantageous for controlling the sensitivity and the dynamic range of LO-based biosensors, demonstrating that the working pH has a powerful influence on kinetics and hence on biosensor operation; at the same time, pH also controls the selectivity of Lys assay [13], thus, care should be applied in pH optimisation.

Finally, it is worth of note that allostery could be also useful for generating sharp, all-or-none responses (*n*_app_ much more than 1), i.e., in enzyme “logical gate” applications where it is necessary e.g., to discriminate between physiological and pathological levels [33]. Unfortunately, LO displays a maximum *n*_app_ = 2, thus, sharper responses are unlikely; in any case, coupling LO with another Lys enzyme displaying different affinity towards Lys (e.g., lysine 2-monooxygenase) could permit steeper input/output responses. This approach could be quite useful in the production of extracorporeal reactor devices to be used to remove pathological Lys levels.

## 4. Conclusions

The present study confirmed the allosteric behaviour of the LO enzyme, as already pointed out in previous studies [15,16]. For LO from *Trichoderma viride*, the allosteric behaviour is strongly dependent on pH and Lys concentration, a feature never reported before. Both Hill and MWC fitting of experimental data showed that cooperativity in LO increases significantly with pH starting from pH 7 up to pH 9; using the Hill approach, the relevant *n*_app_ values increased from about 1 to 2, respectively. When cooperativity is significant, the allosteric behaviour of LO is also dependent from enzyme saturation, i.e., from substrate concentration, ruling out the use of the Hill model to describe the LO kinetics in these conditions. Indeed, the observed Hill plots deviated from the expected linearity, assuming a skewed sigmoidal shape, which agree with the MWC model; accordingly, cooperativity in LO was negligible at low and high enzyme saturations, but maximal at about half enzyme saturation. To take into account this model, the existence of two different conformational states of the enzyme was postulated, which differ in Lys species landing on LO to form the enzyme–substrate complex. This hypothesis was supported also by the distribution of Lys species with pH, which shows the interconversion of the diprotic to monoprotic forms of Lys in the pH range where cooperativity was observed to increase.

The allosteric behaviour of LO can thus be advantageous to control the sensitivity and the dynamic range of LO-based biosensors and, when eventually coupled with another enzyme displaying different affinity towards Lys, useful to produce extracorporeal reactor devices, which gates Lys removal at just above the desired level. Last but not the least, the present study also shows the effectiveness of using an immobilised enzyme and amperometric biosensor not only for substrate analysis, but also as a convenient tool for enzyme kinetic studies.

## Figures and Tables

**Table 1:** Apparent Hill coefficients (*n*_app_) vs. pH at different flow rates.

Flow rate (mL min^−1^)	pH 5	pH 7	pH 9
0.1	1.07 (±0.07)	1.23 (±0.06)	1.93 (±0.20)
0.6	1.01 (±0.05)	1.16 (±0.03)	2.00 (±0.16)
1.0	1.02 (±0.05)	1.13 (±0.04)	1.90 (±0.12)
1.5	1.09 (±0.06)	0.96 (±0.07)	1.77 (±0.09)

Apparent Hill coefficients calculated by fitting the experimental data (20 data points) with the Hill equation; data in brackets are standard errors in apparent Hill coefficient estimations (three replicates).

## Supplementary Materials

The following are available online at https://www.mdpi.com/2079-6374/10/10/145/s1, Scheme S1. Kinetic scheme of an amperometric enzyme electrode (biosensor) showing (left to right) the electrode surface, the enzyme entrapping membrane and the substrate supplying solution. Straight arrows refer to the mass flow of substrate (S) and product (P), while curved arrows refer to the reaction with enzyme (E) and with electrode surface generating electrons (e^−^). Shading refers to substrate concentration gradient in solution and membrane, Scheme S2. Distribution diagram (i.e., dissociation degree **α** vs. pH) for L-lysine species in solution. The dissociation degrees for each L-lysine species have been calculated assuming p*K*_a_ values of 2.2, 8.9 and 10.3, Figure S1: Hill plots of normalised current responses due to injections of L-lysine standard solutions at the amperometric enzyme electrode as a function of L-lysine concentration at pH 9 at different flow rates, as pointed out in the legend. Experimental conditions are described in the Materials and Methods section.
