# Supplementary material for: Allosteric Enzyme-Based Biosensors—Kinetic Behaviours of Immobilised L-Lysine-α-Oxidase from Trichoderma viride: pH Influence and Allosteric Properties
Source: Biosensors (Basel). 2020 Oct 17;10(10):145. doi: 10.3390/bios10100145 (PMC7603024; doi:10.3390/bios10100145)
Supplement: Supplementary file 1 [file biosensors-10-00145-s001.pdf]

biosensors

*Article*

# Allosteric Enzyme-Based Biosensors—Kinetic Behaviours of Immobilised L-Lysine-$\alpha$-Oxidase from *Trichoderma viride*: pH Influence and Allosteric Properties

**Antonio Guerrieri, Rosanna Ciriello [1], Giuliana Bianco, Francesca De Gennaro and Silvio Frascaro**

## Supplementary Materials

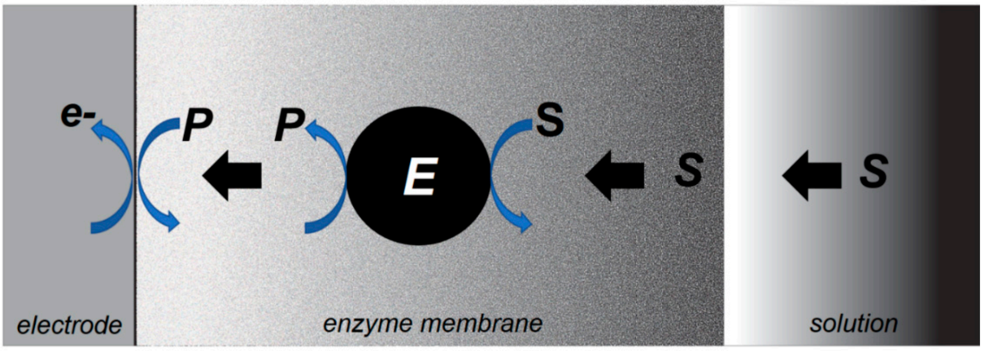


**Scheme S1.** Kinetic scheme of an amperometric enzyme electrode (biosensor) showing (left to right) the electrode surface, the enzyme entrapping membrane and the substrate supplying solution. Straight arrows refer to the mass flow of substrate (S) and product (P) while curved arrows refer to the reaction with enzyme (E) and with electrode surface generating electrons ($e^-$). Shading refers to substrate concentration gradient in solution and membrane.

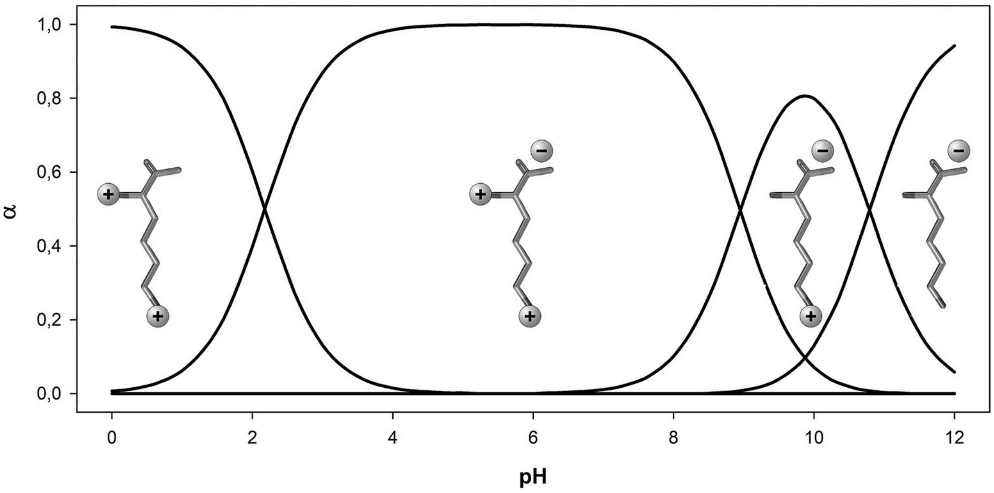


**Scheme S2.** Distribution diagram (*i.e.* dissociation degree $\alpha$ vs pH) for L-lysine species in solution. The dissociation degrees for each L-lysine species have been calculated assuming p$K_a$ values of 2.2, 8.9 and 10.3.

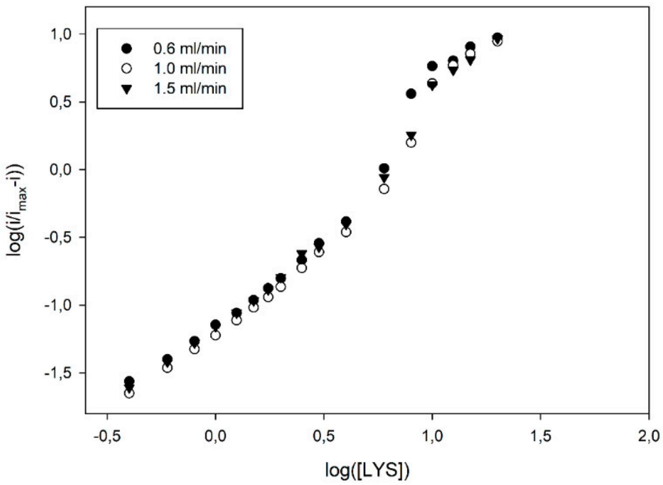


**Figure S1.** Hill plots of normalised current responses due to injections of L-lysine standard solutions at the amperometric enzyme electrode as a function of L-lysine concentration at pH 9 at different flow rates as pointed out in legend. Experimental conditions as described in Materials and Methods section.

© 2020 by the authors. Licensee MDPI, Basel, Switzerland. This article is an open access article distributed under the terms and conditions of the Creative Commons Attribution (CC BY) license (http://creativecommons.org/licenses/by/4.0/).
